# The use of columns of the zeolite clinoptilolite in the remediation of aqueous nuclear waste streams

**DOI:** 10.1007/s10967-018-6329-8

**Published:** 2018-11-22

**Authors:** Alan Dyer, Joe Hriljac, Nick Evans, Ian Stokes, Peter Rand, Simon Kellet, Risto Harjula, Teresia Moller, Zoe Maher, Ross Heatlie-Branson, Jonathan Austin, Scott Williamson-Owens, Manon Higgins-Bos, Kurt Smith, Luke O’Brien, Nick Smith, Nick Bryan

**Affiliations:** 1 Institute of Materials Research, University of Salford, Salford, M5 4WT UK; 2 School of Chemistry, University of Birmingham, Edgbaston, Birmingham, B15 2TT UK; 3 Fakulta jaderná a fyzikálně inženýrská, České vysoké učení technické v Praze, Břehová 78/7, 115 19 Staré Město, Praha 1, Czech Republic; 4 Sellafield, Seascale, Cumbria CA20 1PG UK; 5 Laboratory of Radiochemistry, University of Helsinki, Helsinki, Finland; 6 National Nuclear Laboratory, Central Laboratory, Sellafield, Seascale, Cumbria CA20 1PG UK; 7 National Nuclear Laboratory, Chadwick House, Warrington Road, Birchwood Park, Warrington, WA3 6AE UK; 8 School of Earth Atmospheric and Environmental Sciences, University of Manchester, Oxford Road, Manchester, M13 9PL UK; 9 Department of Chemistry and Forensics, Nottingham Trent University, Erasmus Darwin Building, Clifton Campus, Nottingham, NG11 8NS UK

**Keywords:** Ion exchange, Clinoptilolite, Caesium, Strontium, Effluent treatment

## Abstract

Mud Hills clinoptilolite has been used in an effluent treatment plant (SIXEP) at the Sellafield nuclear reprocessing site. This material has been used to remove ^134/137^Cs and ^90^Sr successfully from effluents for 3 decades. Samples of the zeolite have been tested in column experiments to determine their ability to remove radioactive Cs^+^ and Sr^2+^ ions under increasing concentrations of competing ions, Ca^2+^, Mg^2+^, Na^+^ and K^+^. These ions caused increased elution of Cs^+^ and Sr^2+^. Ca^2+^, Mg^2+^ and K^+^ were more effective competitors than Na^+^. For Na^+^, it was found that if concentration was reduced, then column performance recovered rapidly.

**Electronic supplementary material:**

The online version of this article (10.1007/s10967-018-6329-8) contains supplementary material, which is available to authorized users.

## Introduction

Ion exchange provides a reliable and cost-effective method for removing radioactive Cs^+^ and Sr^2+^ from liquid wastes, provided that the ionic strength is relatively low [1]. In 1984, BNFL Ltd. published details of the site ion exchange effluent plant (SIXEP) on their Sellafield site, which uses columns of the ion exchanger clinoptilolite to remediate alkaline nuclear fuel storage pond waters and other legacy high pH aqueous nuclear waste streams [2]. Clinoptilolite, a naturally occurring zeolite, can exchange Cs^+^ and Sr^2+^ radioisotopes selectively from aqueous solutions in the presence of other common cations, particularly Na^+^ [3, 4]. For the clinoptilolite used at Sellafield, 1 mol of Sr^2+^ and 20 mol of Cs^+^ may be selectively extracted in the presence of 7.5 × 10^5^ mol of Na^+^, 6.5 × 10^3^ mol of Mg^2+^ and 5 × 10^3^ mol of Ca^2+^ [5].

Clinoptilolite is one of the most naturally abundant zeolites [6]. It is mined commercially in at least 16 countries, so ensuring its supply. Further, as an aluminosilicate, it is compatible with encapsulation for long-term storage and subsequent disposal [7]. It can also be used in particle sizes appropriate for columns, and most clinoptilolites are stable under the conditions encountered in nuclear effluents. During the design of the SIXEP plant, there was an extensive evaluation of a wide range of inorganic and organic cation exchange materials. The study recommended that clinoptilolite from the Calico Hills formation at Mud Hills, near Edwards Air Base in California, be selected, following comparison with other clinoptilolite from deposits in Turkey, Japan, South Africa and the USA. Ames [8] studied the performance of several natural zeolites (clinoptilolite, mordenite, chabazite, stilbite, analcite, sodalite) and a synthetic sample of Zeolite A. Although the synthetic sample showed the highest selectivity for Sr^2+^ over Ca^2+^, clinoptilolite was the best performing of the natural zeolites. Marinin and Brown [1] tested the selectivity of a range of natural and synthetic zeolite ion exchangers, ion exchange resins and composite materials. They found that in the low Ca concentration region (< 20 ppm), clinoptilolite showed the second highest selectivity for Sr^2+^ over Ca^2+^ (the best performing material was a synthetic carbon fibre material, impregnated with MnO_2_), even though the clinoptilolite had a relatively low K_d_ compared to some of the other materials. Interestingly, the natural clinoptilolite showed better selectivity than synthetic zeolites. At higher Ca concentrations, the clinoptilolite was outperformed by other materials, but such concentrations are not observed in the feeds to SIXEP.

Clinoptilolite is one of 20 naturally occurring zeolites that have been used to extract aqueous radionuclides, and although Cs^+^ and Sr^2+^ are the most commonly extracted species, U, Th, Ra, Am, ^3^H and Co are also extracted [5, 9]. It has been suggested that clinoptilolite could form a component of permeable reactive barriers for the remediation of Cs^+^ and Sr^2+^ contaminated sites [10, 11]. Valcke et al. [12, 13] have suggested the use of clinoptilolite as a soil amendment to prevent uptake by plant roots. An extensive review of the use of clinoptilolite, and other zeolites, to scavenge radioisotopes is available [14]. A further general review of zeolite ion exchange covers cation selectivity series and details of the theoretical basis of their action [5].

### Clinoptilolite structure and exchange sites

The clinoptilolite used in SIXEP has an ideal formula of Na_6_Al_6_Si_30_O_72_·24H_2_O. This gives a theoretical cation exchange capacity (CEC) of 2.2 meq (milli-equivalents) per gram. However, the typical effective CEC of the clinoptilolite used in SIXEP is less than this, as it has an empirical formula of Na_2.20_Mg_0.40_Ca_0.99_Sr_0.07_Al_6_Si_30_O_72_·24H_2_O. The SIXEP clinoptilolite contains 95% clinoptilolite, with smaller amounts of albite, barite, mica, gypsum, dolomite, *K*-feldspar, quartz, magnetite, illite and montmorillonite, which are included in the empirical formula [15]. The presence of Group II cations reduces the effective CEC, because Ca^2+^, Mg^2+^ and Sr^2+^ are not exchanged as readily as Na^+^.

Clinoptilolite has narrow channels with pore diameters in the range of 3.5–3.9 A, which is similar to the size of a hydrated Cs^+^ ion [16]. This may be responsible for the high Cs^+^ selectivity over other monovalent species. Three distinct cation binding sites are found within the clinoptilolite structure: Na1, Ca2, K3. The Na1 site has ninefold coordination, whilst the Ca2 and K3 sites are characterised by eightfold coordination [17]. Um and Papelis [17] used X-ray absorption spectroscopy to probe the coordination environment of Sr^2+^ in clinoptilolite. They found that the oxygen coordination was 8-coordinate. The spectra were like those of Sr^2+^(aq), and so they describe the Sr^2+^ binding as ‘outer sphere’, but there was a small reduction in coordination number compared to hydrated Sr^2+^, which could be associated with a loss of some water. The spectra were consistent with coordination by 8 oxygens at a distance of 0.26 nm ± 0.002 nm, which is consistent with the Ca2 site (CN 8, 0.258 nm). Although the K3 site is also eight coordinate, the bond distance is longer (0.302 nm). Ca^2+^ can also occupy the Na1 site, and so Um and Papelis [17] suggested that Sr^2+^ might also do this, but the data suggested that occupation of this site was not significant for their sample, which could be due to the larger size of Sr^2+^ compared to Ca^2+^. O’Day et al. [18] used Extended X-ray absorption fine structure (EXAFS) spectroscopy to study the Sr^2+^ binding sites in heulandite samples. They found 3 distinct Ca^2+^ positions in the zeolite channels, each with 5 waters coordinated to each Ca, with additional coordination by 3 oxygen atoms from the heulandite lattice. Using geometrical arguments, Palmer and Gunter [15] have suggested that Ca^2+^, Mg^2+^ and Sr^2+^ must lose part of their hydration shell to travel through the channels in the clinoptilolite structure. For Sr^2+^ loaded samples, the EXAFS spectra of O’Day et al. [18] were consistent with Sr^2+^ coordination by 8 oxygen atoms in the first shell and with two shells of Al/Si, both with approximately 2 atoms. Their data are consistent with Sr^2+^ occupying a Ca position in the lattice. Um and Papelis [17] determined the effect of competing cations on Sr^2+^ sorption, and found that ions were effective in the order Ca^2+^ > Mg^2+^ > Na^+^. They suggest that the closer charge/size match between Sr^2+^ and Ca^2+^ is the reason for the effective competitive effect of Ca.

O’Day et al. [18] predicted that Cs^+^ should occupy the site normally occupied by K^+^. Interestingly, Na^+^ can occupy the same site as Ca^2+^, due to its smaller size, compared to K^+^/Cs^+^, and so direct exchange of Sr^2+^ and Na^+^ is expected [18]. Smyth et al. [6] studied the structure of a Cs^+^ substituted clinoptilolite by single crystal X-ray diffraction, and found that the position occupied by Cs^+^ could not be related to any of the positions of the normal extra-framework exchangeable ions. Further, the Cs-O bonds were long (3.0–3.5 Å). Interestingly, there was evidence for the loss of water from the structure upon ion exchange for Cs^+^. The authors suggested that there was rearrangement of cation sites in the structure rather than simple one for one cation exchange in the case of Cs^+^.

This paper describes column experiments and modelling work that was performed between 1978 and 2012 in support of the design and operation of the SIXEP facility at Sellafield. Despite the extensive literature describing the use of clinoptilolite in nuclear waste remediation, and related areas, there have been few studies on breakthrough curves, although some Cs^+^ and Sr^2+^ data have been reported for Siberian tuffs containing 50–66% clinoptilolite [19] and more recently Nikashina et al. [10] have presented a limited number of Sr breakthrough curves. What follows is a short description of the plant, and its role in effluent processing and the reduction of radioactive discharges to the Irish Sea. Then there is a description of the experimental and modelling programme that was used to assess and predict the performance of the ion exchange material and the plant.

The experimental programme to support SIXEP was performed in two stages. Initial work, prior to the commissioning of the plant, was performed at the Harwell UKAEA laboratories (Oxfordshire, U.K.) between 1978 and 1982. Later, an on-going experimental programme was commissioned (starting in 2004), with laboratory work taking place at the National Nuclear Laboratory (at Sellafield, U.K.), supported by Sellafield Ltd.

### The site ion exchange effluent plant

**Fig. 1:**
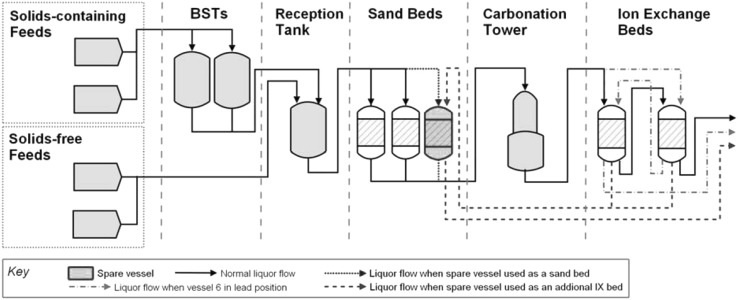
Representation of the SIXEP process

SIXEP is an ion exchange plant that was designed to remove ^137^Cs and ^90^Sr from alkaline liquid effluent. A diagram of the SIXEP plant is shown in Fig. 1. It consists of the following units:

- **Settling tanks** that allow large particulates to settle from feed solutions before they are fed to the sand bed filter;
- Two **sand bed filters** operate in parallel to remove suspended solids to protect the ion exchange beds from blinding and to reduce the soluble [Mg^2+^] in the columns. A quaternary amine polyelectrolyte flocculant is added to the solution prior to the sand bed to improve filtration performance;
- A **carbonation tower**, which adjusts the pH of the solution emerging from the sand bed from ~ 11 to ~ 7 to protect the clinoptilolite beds, which degrade at high pH;
- Two **clinoptilolite beds**, which operate in series (one lead bed and one lag bed). The lead bed is replaced with fresh media when it is exhausted, and the bed that previously operated in the lag position is promoted to the lead position.

The contact time of effluent with the SIXEP clinoptilolite column is approximately 8 min, due to the very high flow rate.

The major feeds into SIXEP are effluents from nuclear fuel storage ponds and the washings from fuel de-canning operations. Contributions also come from other parts of the Sellafield site. Pond water purges account for the bulk of the feed to SIXEP (100 s of m^3^/day) and consist of demineralised water that has been dosed with NaOH to increase the pH of the pond to at least 11. This is done to minimise the potential for corrosion of the fuel by protecting the cladding and to control the solubility of Mg derived from historical corrosion of Magnox cladding (a Mg/Al alloy, which when corroded forms Mg hydroxides and carbonates; [20, 21]). Some of the facilities contain stocks of corroded fuel and Magnox sludge from historical operations, and ^134/137^Cs and ^90^Sr are still released into the water from that material.

Effluent from de-canning operations, flask washing, and sand bed backwashes are routed to the bulk storage tanks (BST), where they are allowed to settle to separate most of the particulates. Water is purged from the ponds to reduce the activity within the pond and provide safe working conditions for operators. The purge is routed to the sand bed filters (via a reception tank, where it is periodically blended with feeds from the BST). The de-canner washings consist of demineralised water and fines from the fuel cladding, as well as some ^137^Cs and ^90^Sr. On average, the BST route accounts for approximately 40% (range 10–80%) of the radioactivity input to SIXEP, though it contributes a relatively much smaller volume.

One of the factors that govern the performance of an ion exchange material is the presence of competing ions. Clinoptilolite is very selective for Cs^+^ and Sr^2+^ over Na^+^, which is required given the high Na concentration in the SIXEP feed solutions. Competing ions that are known to exist in appreciable concentrations in the feeds to SIXEP include:

- Na^+^ (up to 100 ppm) from the NaOH used to maintain a high pH;
- Mg^2+^ (typically < 0.6 ppm) that arises from dissolution of corroded Magnox nuclear fuel cladding;
- Ca^2+^ (typically < 1 ppm) that is naturally present in water supplies and may leach from sludges and concrete infrastructure;
- K^+^ (typically < 1 ppm) an impurity in the NaOH.

## Experimental details

A series of 5 mL column experiments were performed between 1978 and 2012. The same general method was applied in these studies: a typical procedure is described below.

At the start of the experimental programme, a standard simulant feed was defined that was used throughout. It was designed to mimic the chemistry of the fuel storage pond effluents following carbonation. Table 1 shows the composition of this simulant. The simulant was spiked with 25 Bq mL^−1^ of each of ^137^Cs and ^85^Sr (^90^Sr was used in original Harwell experiments between 1978 and 1982). The laboratory column experiments were scaled down versions of the real SIXEP column.

**Table 1:** Harwell simulant composition

	ppm (mg L^−1^)	mmoles L^−1^
Na	100	4.35
Si	3.7	0.13
Ca	1.5	0.04
Cl	5.6	0.16
Mg	1	0.04
Cs	1.7 × 10^−2^	1.2 × 10^−4^
Sr	5.02 × 10^−4^	5.72 × 10^−6^

Due to the nature of the column tests, a smaller clinoptilolite particle size was adopted (420–500 μm) than that used on plant. The reduction in particle size was necessary to reduce edge effects in the columns, i.e., where the feed flows down the walls without making proper contact with the ion exchange material. The clinoptilolite was untreated (as-received) material to match its use on plant. Batches of clinoptilolite were pre-sieved to separate the 420–500 μm fraction. The sieved material was washed to remove fines and dried at 110 °C until a constant weight was achieved. Portions were then slurried into chromatography columns (0.9 cm diameter) to make up a column of 5 mL volume. This was repeated 5 times with different portions of sieved material, which were then dried to a constant weight to determine the average mass of material required to produce a 5 mL column, which was 4.70 g.

The experimental rig consisted of feed tanks from which the simulant was pumped to the 5 mL columns of clinoptilolite in parallel. The active simulant was pumped through the columns using precision peristaltic pumps. A schematic diagram of the rig layout is shown in Fig. A1 (Supporting Information) and a photograph of the rig in Fig. A2 (Supporting Information).

The columns were prepared as follows: a mesh was inserted into the column (to prevent loss of clinoptilolite particulate into the effluent); 3.52 g of glass beads were added to the column and a further mesh added (to separate the glass from the clinoptilolite); 4.70 g of clinoptilolite (sieved and washed) was added and a further mesh added; sufficient glass beads were added to fill the remainder of the column. The glass beads were added to: [i] pack out the column spare space; [ii] provide a means to distribute the flow before it entered the clinoptilolite beds; and [iii] provide a tortuous path for any clinoptilolite particles (i.e. prevent loss of clinoptilolite particles into the effluent). The completed column was a well packed bed of clinoptilolite material with a standard mass (see Fig. A3 (schematic diagram) and Fig. A4 (photograph); Supporting Information).

The flow rate was that of the SIXEP design flow-sheet (4200 m^3^ day^−1^), equivalent to a superfacial velocity of 22 m^3^ m^−2^ h^−1^. For the 5 mL columns, this is equivalent to a flow rate of approximately 20 bed volumes per hour (BV hr^−1^). Samples of the effluent from each column were taken regularly to determine the breakthrough of activity.

Samples of effluent were taken regularly from each column. The volume of solution collected over a 1 h period was measured to confirm that it was being fed at the required flow rate. Effluent pH was in the range of pH 7.6–8.6. The temperature during the experiments was not controlled, because that was not feasible given the restrictions involved in the use of radioactive materials and because of the very long duration of the trials (several months). Therefore, the experiments were carried out at room temperature, with variation from day to day and overnight. The temperature in the laboratory during the experiments was typically in the range 16–22 °C. The first phase experiments were also performed at room temperature, but the temperature was not recorded at the time. The variation in flow rate was < 15% (For 2nd phase experiments).

The column effluent was prepared for gamma spectrometry to determine the concentrations of ^137^Cs and ^85^Sr. A 50 mL portion of the column effluent was transferred into a pre-prepared 125 mL vial, which contained 0.1 mL of 10 g L^−1^ Cs^+^ (as CsNO_3_), 0.1 mL of 10 g L^−1^ Sr^2+^ [as Sr(NO_3_)_2_] and 0.1 mL of 2 M HNO_3_ as carriers to stabilise the sample (to prevent sorption of activity onto the surface of the vial). The stabilised samples were counted for 3600 s by gamma spectrometry as soon as possible (Eγ(^85^Sr) = 514.0 keV; Eγ(^137^Cs) = 661.7 keV). Samples of the feed liquor were also collected each time a new batch was prepared to verify that it had been correctly dosed with ^85^Sr and ^137^Cs. The gamma spectrometry system comprised of several Na iodide detectors. The system was calibrated using solutions of the same geometry as the effluent and feed samples, with known activities of ^85^Sr and ^137^Cs.

### First phase Harwell experiments (1978–1982)

Prior to the commissioning of SIXEP, many 5 mL column experiments were performed.

Many boreholes were drilled at the Mud Hills site to obtain batches of clinoptilolite (details below). A sub-sample of each batch was used in a 5 mL column experiment using the standard Harwell simulant (Table 1). One sample of this material was identified as a ‘standard’ clinoptilolite, and material from this borehole was then characterised in column experiments that used variants of the Harwell standard simulant. These included concentrations in the following ranges:

- Cs^+^, 0.02 mM–0.17 mM;
- Sr^2+^, 0.92 µM–9.2 µM;
- Ca^2+^, 0 mM–0.15 mM;
- Mg^2+^, 0.02 mM–0.08 mM;
- Na^+^, 2.17 mM–8.69 mM.

### Second phase experiments (2007–2012)

The second phase experiments used the same general method as that for the first phase. However, clinoptilolite from a different borehole was used. 5 mL column experiments were used to study the following:

- Behaviour with standard Harwell simulant.
  The behaviour of Cs^+^ and Sr^2+^ in standard Harwell simulant (Table 1) was determined.
- Effect of K concentration
  Known concentrations of K^+^ ions (as aliquots of a 5000 ppm KCl stock) were added to the standard simulant to give concentrations of 1 or 5 ppm (0.024–0.12 mM). In addition, experiments were performed with the Harwell simulant with no added K^+^.
- Effect of pulsed high Na concentrations
  Triplicate column experiments were run in which the concentration of Na in the feed alternated between 100 and 250 ppm (4.35 and 10.8 mM), otherwise the chemistry was that of the Harwell simulant. The Na pulsing was initiated 1 week after the trials started, i.e. after approximately 3500 BV had been processed. The Na pulsing was conducted each week for the next 6 weeks of the trial, with the high Na simulant being fed through for 3 days per week. For the rest of the week, the standard Harwell recipe was injected. After 6 weeks, the simulant pulsing was stopped and the columns were fed with the unaltered Harwell simulant to the end of the experiment.

### Batch experiments

*K*-form clinoptilolite was prepared by eluting KCl solution (2 M; 2 L) through a column (4 g clinoptilolite). The K-form material was found to have the composition (meq/g): *K* = 1.9; Na = 0.097; Mg = 0.082; Ca = 0.030. Batch exchange experiments were performed using the K-form clinoptilolite by equilibrating 0.08 g of zeolite with 8 mL of 0.01 M, 0.1 M and 1 M solutions of KNO_3_. The initial concentration of ^85^Sr in the experimental solutions was approximately 700 Bq mL^−1^. At intervals, the samples were centrifuged (30 min; 20,000 rpm) and filtered through a 0.22 µm filter. Identical experiments were performed with the same solid:solution ratio, but in the presence of 1 mM of bicarbonate (KHCO_3_). The activities of the solutions were determined by gamma spectrometry. The equilibrium was followed by periodic sampling, and the reactions were at equilibrium after 3 weeks. At this point, the distribution coefficients (*K*_*d*_) were calculated using (1):

1 $$K_{d} \left( {{\text{ml}}/{\text{g}}} \right) = \frac{{concentration \,of\, radionuclide\, on\, solid \left( {Bq/{\text{g}}} \right)}}{{concentration\, of\, radionuclide \,in \,solution \left( {Bq/{\text{ml}}} \right)}}$$

## Results and discussion

The experimental data presented in the figures are available in the electronic supporting information. The effectiveness of the zeolite was measured by plotting the quantity of Cs^+^ and Sr^2+^ in the effluent as a percentage compared to that in the feed versus the number of bed volumes of solution treated. Interpretations of such elution curves are typically based on the selectivity that clinoptilolite shows for the cations studied, as determined from ion exchange isotherms [5], i.e.

$$Cs >K >Sr =Ba>Ca\gg Na>Li\,[22]$$

$$Ba \approx Sr > Ca > Mg\,[3]$$

It should be noted that, according to Eisenman’s Theory [23], clinoptilolite, as a high silica zeolite, should show a preference for large monovalent cations (e.g., Cs^+^, K^+^), rather than smaller monovalent or divalent cations. Zhao et al. [24] studied a series of synthetic clinoptilolite-like zeolites, and found that increasing the Al content of the material did improve uptake of divalent ions. Thus, the unusual ability of clinoptilolite to scavenge Sr from aqueous nuclear wastes has been an obvious bonus to the nuclear industry and has led to speculation that it exists as a speciated monovalent cation [25, 26], such as [SrHCO_3_]^+^. This will form part of later discussions.

The interpretation of the column experiment results and the performance of SIXEP is further complicated by the very short residence time of the solutions in the columns. Ames [27] studied the kinetics of Cs^+^ uptake by Californian clinoptilolite samples, and found that Li^+^ substituted material gave the fastest uptake, followed by Na^+^ and K^+^, with Ca^2+^, Ba^2+^ and H^+^ substituted material showing slower exchange. Shahwan et al. [28] studied the uptake of Cs^+^ by clinoptilolite. They found that the reaction was slow, taking more than 1000 min for equilibrium. The uptake was consistent with a 2nd order kinetic model. Second order Cs^+^ kinetics were also observed by Cortés-Martínez et al. [16]. Shahwan et al. [28] suggested that the uptake kinetics were consistent with a relatively rapid uptake of Sr^2+^ to the surface of the clinoptilolite, followed by slower diffusion into the channels. Faghihian et al. [29] showed that the uptake of both Cs^+^ and Sr^2+^ by natural clinoptilolite was reversible.

Palmer and Gunter [15] analysed the clinoptilolite used in SIXEP. They studied the uptake of Sr^2+^ in batch experiments, and found that it took 10 days for complete equilibrium to be achieved. They also tested the exchange of cations using high concentrations of Sr^2+^, and found that it was effective in displacing Ca^2+^ (98% exchange), but that only 37% of K^+^, 75% of Na^+^ and 56% of Mg^2+^ were displaced. Similarly, Woods and Gunter [30] studied Cs^+^ exchange in SIXEP clinoptilolite, and found that the exchange was quicker than that for Sr^2+^, with most exchange taking place within 10 h, and for Na^+^ and K^+^, most of the exchange was complete within 30 min. At apparent equilibrium, there was exchange of 85% of the available Na^+^, compared to 50% for K^+^, 80% for Ca^2+^, and 40% for Mg^2+^. Therefore, given the very short residence time in the columns and SIXEP (8 min), the exchange reactions for Cs^+^ and particularly Sr^2+^ do not approach equilibrium. As a result, the behaviour of the ions in the columns will be controlled to some extent by the relative exchange kinetics of the ions, probably at the surface of the clinoptilolite. Such initial uptake kinetics are not necessarily in the same order as that of the selectivity that is observed at equilibrium [31].

### First phase (Harwell) experiments

**Fig. 2:**
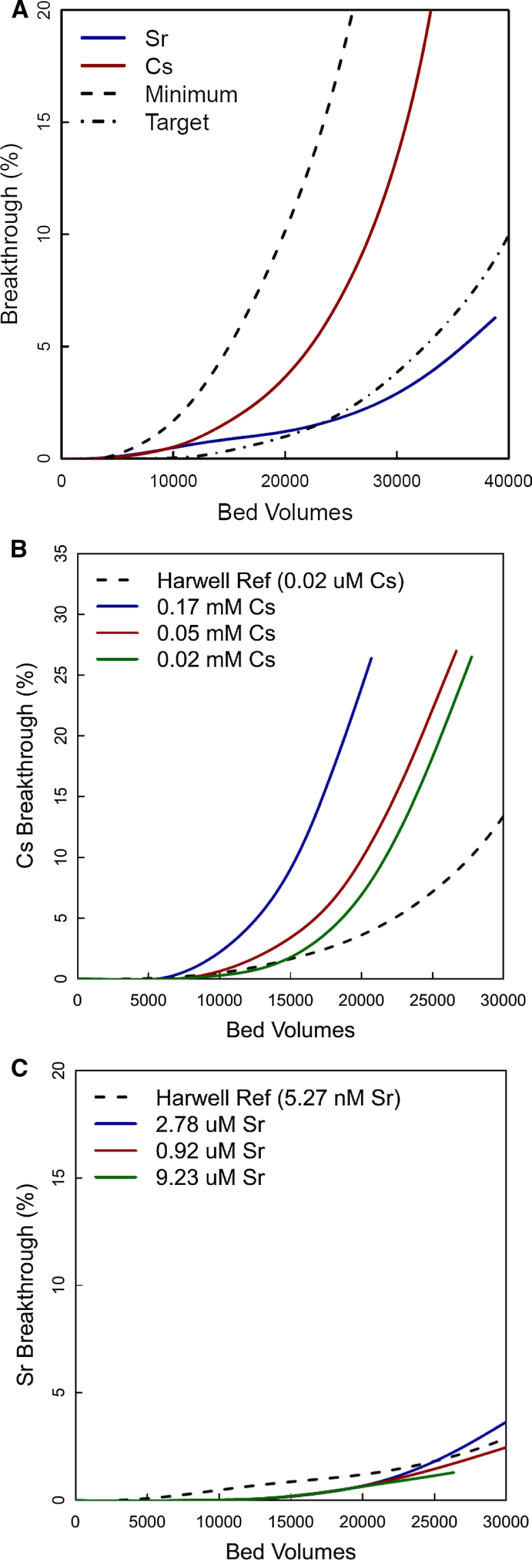
Top—base case breakthrough of Cs^+^ (1.24 x 10^−7^ M) and Sr^2+^ (5.56 x 10^−9^ M). Middle and bottom—effect of Cs^+^/Sr^2+^ concentration on Cs^+^/Sr^2+^ breakthrough

Samples of clinoptilolite from the same borehole were selected for use in the 1978–1982 studies with a range of competing ion concentrations. These would later be used for the calibration of the plant process model. Because of the high capacity of the clinoptilolite for Cs^+^ and Sr^2+^, experiments were not carried through to complete breakthrough (i.e., effluent concentration equal to feed), because of the time required, and because the real ion exchange columns in SIXEP are not operated through to complete breakthrough. Instead, the column experiments ran until an initial breakthrough was observed, which was typically by 30 kBV. Figure 2a shows Cs^+^ and Sr^2+^ breakthrough curves for an experiment using the standard conditions (Table 1). These curves were adopted as the standard performance for SIXEP clinoptilolite, and are referred to as the ‘Harwell Reference’ in the text below. In the original Harwell development work in the 1970′s, minimum and target clinoptilolite performances were defined (Fig. 2a). These were arbitrary elution profiles, based on what could be realistically achieved, i.e., an ideal performance (the target) and a minimum performance that would still be viable, given the cost of disposing of spent clinoptilolite via encapsulation and eventual geological disposal. The performance of the clinoptilolite sample was within the acceptable range for both Cs^+^ and Sr^2+^.

#### Effect of Cs^+^ and Sr^2+^ concentrations

Experiments were performed with raised Cs^+^ concentrations of 0.02, 0.05 and 0.17 mM, and Sr^2+^ concentrations of 0.92, 2.78 and 9.23 μM (Fig. 2b, c). The increased level of Cs^+^ in the 0.17 mM system reduced the number of BV before Cs breakthrough occurs. The breakthrough curves produced for the 0.02 and 0.05 mM Cs^+^ experiments were within the experimental reproducibility (± 10%). There was a reduction of 21% from 21.5 to 15 kBV, when comparing the number of BV required for 10% Cs^+^ breakthrough in the 0.02 and 0.17 mM experiments. All the systems with raised Cs^+^ concentrations showed early breakthrough compared to the ‘Harwell Reference’ profile (Fig. 2b).

Unlike the Cs^+^ experiments, increasing Sr^2+^ concentration in the feed (Fig. 2c) has little effect on the breakthrough up to 30,000 BV. This was due to the superior performance of this sample of ion exchanger with Sr^2+^ in comparison to Cs^+^ (Fig. 2b). In addition, Sr^2+^ concentrations were much lower than Cs^+^ in the experiments (mM vs. µM). This meant that, in contrast to the Cs^+^ experiments, the exchanger was not approaching saturation towards the end of the experiment at any of the concentrations used.

#### Effect of Ca^2+^ and Mg^2+^ concentration

**Fig. 3:**
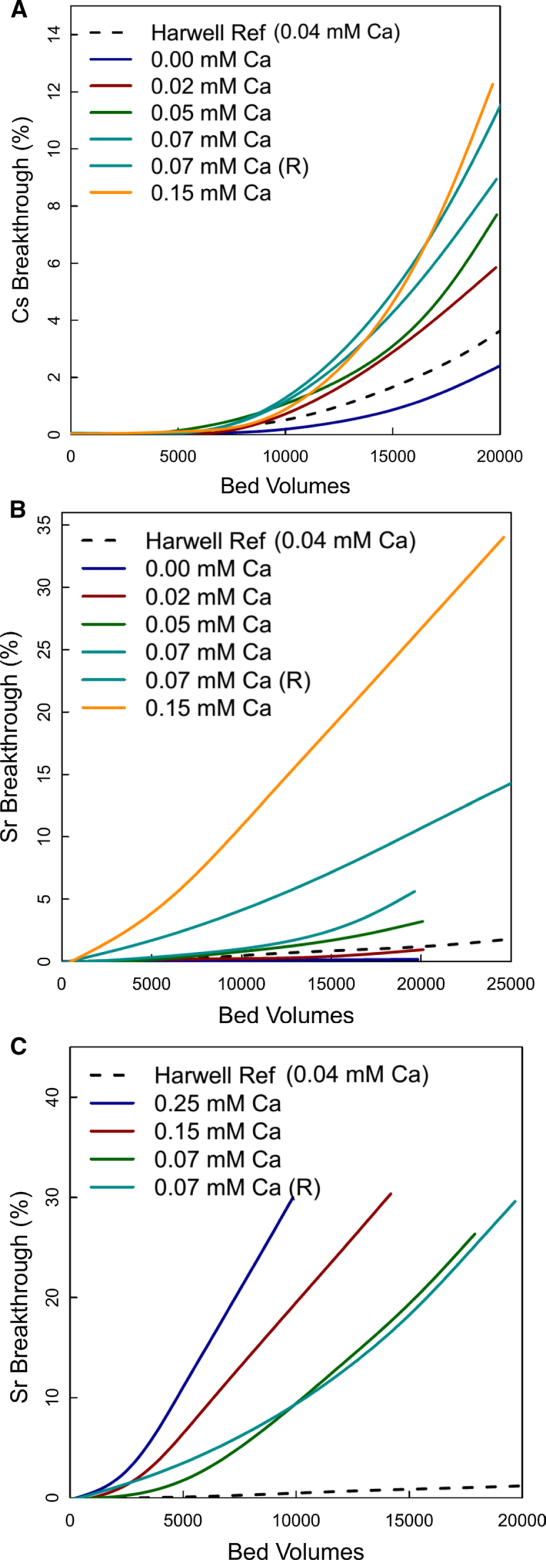
Top and middle—effect of Ca concentration on Cs^+^ and Sr^2+^ breakthrough at [Mg] = 0.02 mM (0.6 ppm); bottom—effect of Ca concentration on Sr^2+^ breakthrough at [Mg] = 2.0 ppm

Figure 3a, b shows the effect of increasing Ca concentration on Cs^+^ and Sr^2+^ breakthrough at a fixed concentration of Mg (0.6 ppm = 0.02 mM). When no Ca was present in the feed, there were breakthroughs of 2.5% Cs^+^ and 0.2% Sr^2+^ after 20 kBV. These were better than the standard breakthrough curve, which had a Ca concentration of 0.04 mM. With 0.02 mM of Ca present, the breakthrough values after 20 kBV have increased to 6% and 1% for Cs and Sr, respectively. When the concentration was increased to 0.05 mM in the feed, the breakthrough values for Cs and Sr after 20 kBV were 8.4% and 3.3%. When the Ca concentration had increased to 0.15 mM, the breakthrough at 20 kBV was 12% and 25% for Cs^+^ and Sr^2+^.

Figure 3c shows the effect of increasing Ca concentration on breakthrough at a fixed Mg concentration (2.0 ppm = 0.08 mM). The repeat data (0.07 mM Ca^2+^) showed good agreement. The data (Fig. 3c) showed the expected trend, with earlier breakthroughs as Ca concentration increased. Further, increasing the Mg^2+^ concentration also had the expected effect, and for systems with identical Ca concentrations, earlier breakthrough was observed at the higher Mg^2+^ concentration (compare the positions of plots with 0.07 and 0.15 mM Ca^2+^ in Fig. 3b, c).

Repeat experiments are shown in Fig. 3 for the 0.07 mM Ca concentration, which gives an indication of the experimental uncertainties. Although the repeat curves were close for the Cs^+^ data in Fig. 3a and the Sr^2+^ data in Fig. 3c, there was a greater difference for the Sr^2+^ experiments shown in Fig. 3b. The reason was unclear and could be due to problems associated with the column experiment or due to the natural variability of clinoptilolite (See below). The order of the profiles in Fig. 3 follows that expected from the zeolite selectivity series, apart from the relative positions of the Harwell reference curves (0.04 mM Ca) and the 0.02 mM Ca plot in Fig. 3a. Given the magnitudes of the differences between the repeat experiments in Fig. 3b, this may be a result of experimental uncertainties and natural variations in the zeolite composition.

**Fig. 4:**
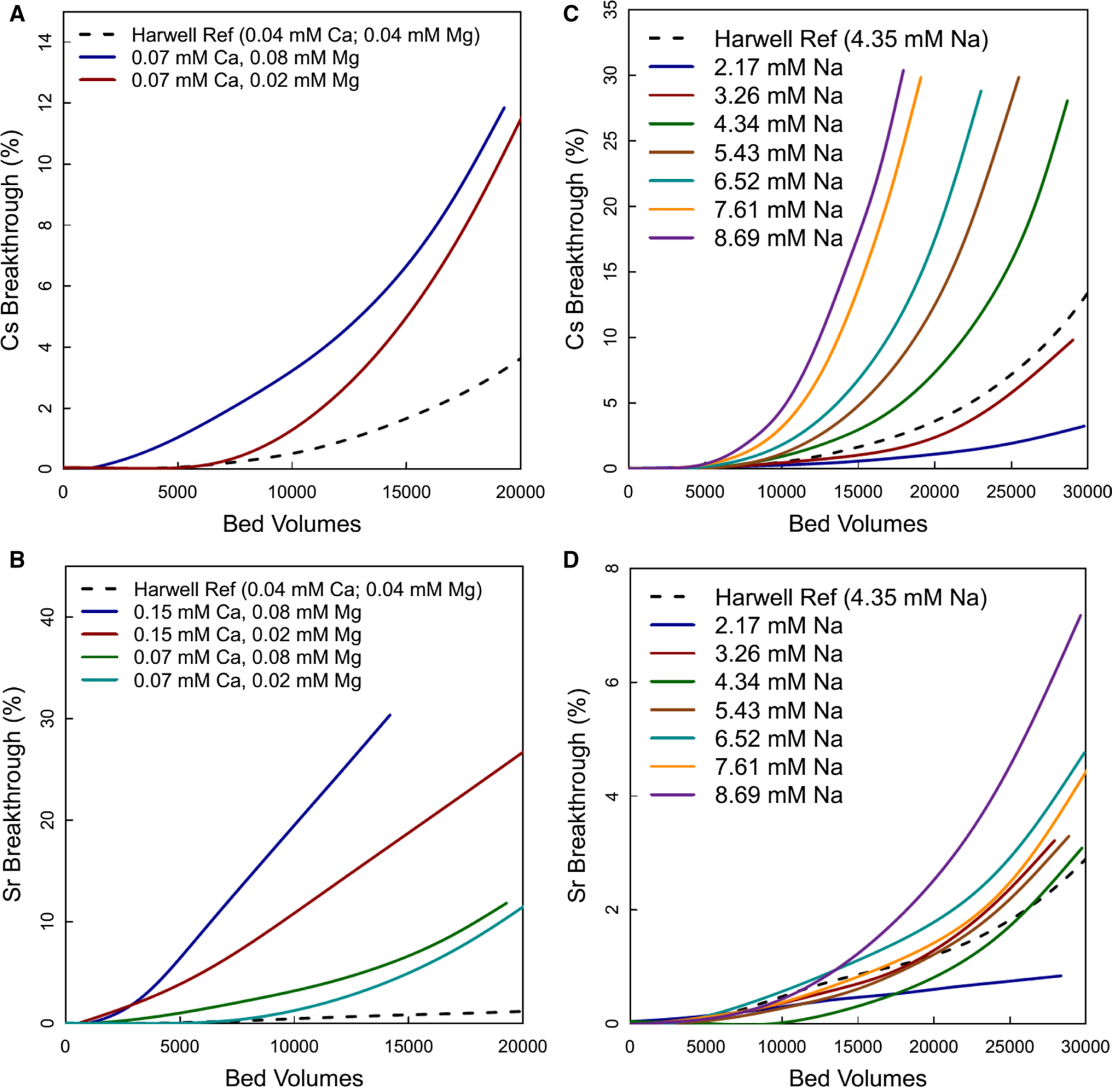
Effect of [Mg^2+^] on Cs^+^/Sr^2+^ breakthrough at [Ca^2+^] = 0.15 and 0.074 mM (3 and 6 ppm)—left hand plots; effect of [Na^+^] on Cs^+^/Sr^2+^ breakthrough (right hand plots)

Figure 4a shows the effect of Mg^2+^ concentration (0.02 and 0.08 mM), at a fixed Ca concentration of 0.07 mM, on Cs^+^ breakthrough. The effect of Mg^2+^ concentration on Cs^+^ breakthrough was as expected, with earlier breakthrough at the higher Mg^2+^ concentration. Figure 4b shows the effect of Mg^2+^ concentration on Sr^2+^ breakthrough, this time at Ca concentrations of 0.07 and 0.15 mM. Qualitatively, the combined Ca^2+^ and Mg^2+^ concentrations had a greater effect on Sr^2+^ than on Cs^+^ (compare difference between plots in Fig. 4a, b and the Harwell Reference curves). The plots in Fig. 4a, b follow the expected trend, with earlier breakthroughs observed as Mg and Ca concentrations increase. As would be expected, comparing Figs. 3 and 4, the effects of Ca^2+^ and Mg^2+^ were additive.

Given the similarity in the binding sites of Ca^2+^ and Sr^2+^ in clinoptilolite (see above; [17, 18]), it is not surprising that Ca^2+^ is very effective at suppressing Sr^2+^ uptake. However, Mg^2+^ seems to be almost as effective as a competing ion. Given the difference in charge density between Cs^+^ and Ca^2+^/Mg^2+^, we would expect the Group II ions to have less effect on Cs^+^ than for Sr^2+^, and this seems to be the case. However, Ca^2+^ and Mg^2+^ can suppress binding to some extent, Smyth et al. [6] has suggested that Cs^+^ exchange is more complex than simple one for one substitution (see above), and this may explain the observations.

#### Effect of Na^+^ concentration

Experiments were performed with seven different concentrations of Na. The breakthrough curves for Cs and Sr for these tests are shown in Fig. 4c, d. The curves with 4.34 mM Na^+^ were effectively repeats of the Harwell reference curves. For the Cs^+^ data, the curves were in the expected order, with progressively earlier breakthrough as Na concentration increased. The difference between the Harwell reference curve and the 4.34 mM Na curve was probably due to natural variation in the clinoptilolite and experimental uncertainty (see below). As would be expected, due to competition effects, breakthrough occurs earlier with increasing Na^+^ concentration, although higher concentrations in comparison to Mg^2+^ and Ca^2+^ were required to have an effect, which would be expected given the similarities between the Ca^2+^ and Sr^2+^ binding sites [17, 18].

Generally, the same concentrations of Na had a smaller effect on Sr^2+^ than Cs^+^. Given the variation in behaviour for the 4.34 mM experiment and the Harwell reference curve, for Cs^+^ it seems that the differences in behaviour below Na concentrations of 8.69 mM Na were probably not significant.

#### Clinoptilolite variability

**Fig. 5:**
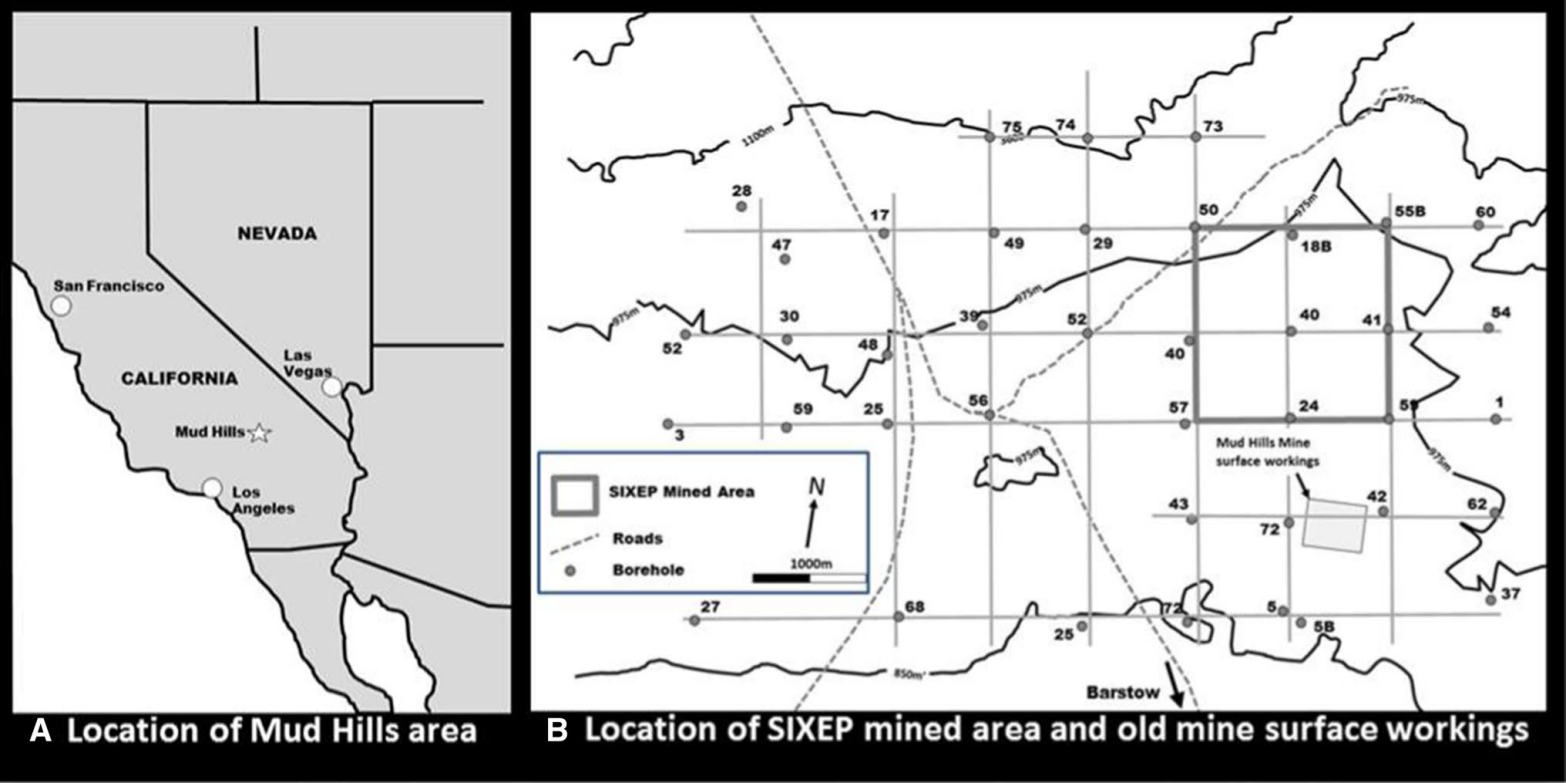
Map of the Mud hills clinoptilolite showing the location of the sample boreholes

During the initial characterisation work, samples from several boreholes across the clinoptilolite deposit were analysed in 5 mL columns to assess their variability. Their location is shown in Fig. 5. Column experiments, all using the standard Harwell simulant recipe (Table 1), were used to identify that part of the deposit that should be mined for use in SIXEP.

**Fig. 6:**
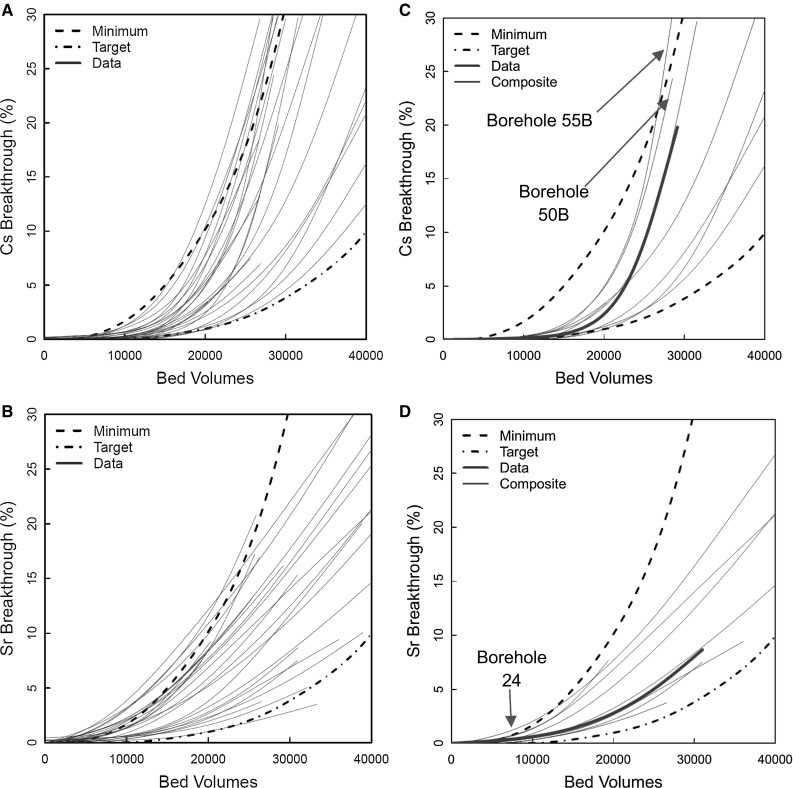
Sr^2+^/Cs^+^ column breakthrough profiles collected from each borehole in the Mudhills mine (left hand plots); profiles for samples from the SIXEP mined area (right hand plots)

Figure 6a, b shows the Cs^+^ and Sr^2+^ breakthrough data for each of the boreholes. There was significant variability in the behaviour of the clinoptilolite obtained from the various boreholes at Mud Hills. Very few of the samples corresponded to the performance reported for the Harwell Reference Curves (Fig. 2a).

The worst performing zeolite samples failed to meet the minimum performance curves for both Cs^+^ and Sr^2+^. At the other extreme, the best performing samples were comparable with the Harwell reference curve for Sr^2+^, and significantly better than the Harwell reference curve for Cs^+^. In fact, the sample used for the Harwell reference curve represented optimum behaviour for Sr^2+^ retention for the deposit, and was not representative of most of the material.

Based on the data in Fig. 6a, b, an area of the site was identified as a source of clinoptilolite for SIXEP, which is shown as a green square in Fig. 5 (boreholes 50B, 18B, 55B, 40, 41, 57, 24 & 58). The mined area was of sufficient size to provide enough material for twice the expected lifetime requirement of SIXEP. Figure 6c, d shows the breakthrough profiles for samples from the mined area for Cs^+^ and Sr^2+^. Even in this smaller area, considerable variability in performance for Cs^+^ and Sr^2+^ was observed. Despite the variability, nearly all the samples fell between the minimum and target performances. There were two exceptions. The Sr^2+^ curve for borehole 24 failed to meet the minimum performance values at low BV, but was within target and minimum after ~ 13 kBV (representing breakthrough of up to 3%). For the Cs^+^ curves, borehole 55B exceeds the minimum curve above approximately 26 kBV, and by extrapolation, borehole 50B would have exceeded the minimum curve at ~ 29 kBV if the experiment had continued. These excursions outside the minimum performance were acceptable, as the number of BV to which the clinoptilolite is exposed in SIXEP rarely exceeds 25,000.

A composite sample was made from a combination of the material from each borehole in the SIXEP mined area (Fig. 5). This composite sample was then used in column experiments. The resulting breakthrough curves are shown in Fig. 6c, d. The composite samples fell between the minimum and target breakthrough profiles for both Cs^+^ and Sr^2+^, although neither displayed a performance comparable to the Harwell Reference curves.

Most samples displayed an ion exchange preference for either Cs^+^ or Sr^2+^, and in doing so were poorer exchangers for the other ion. This was evident in the mined SIXEP area data, e.g., borehole 58 gave one of the best ion exchange profiles for Cs^+^, but one of the worst profiles for Sr^2+^. The exceptions to this, where there was no strong preference for either ion, were boreholes 49 and 29, and to a lesser extent 17 and 47. It seems that samples from the North of the SIXEP mined area showed a preference for Sr^2+^, whilst those from the South preferred Cs^+^. No chemical analyses were performed at the time of the work, but selectivity would be expected to depend upon the Si/Al ratio of the clinoptilolite (see above; [23]).

### Phase 2 experiments

Approximately 25 years after the initial work to characterise the ion exchange performance of the clinoptilolite, a further series of column tests were initiated. These used clinoptilolite from a different location to that used in the earlier tests. Given the variability in the performance of the material across the mined area (Fig. 6c, d), the baseline performance of the material to the standard feed simulant would be expected to be different, but the same general trends would be expected: e.g., earlier breakthrough with increasing competing ion concentration.

#### Effect of K^+^ concentration

**Fig. 7:**
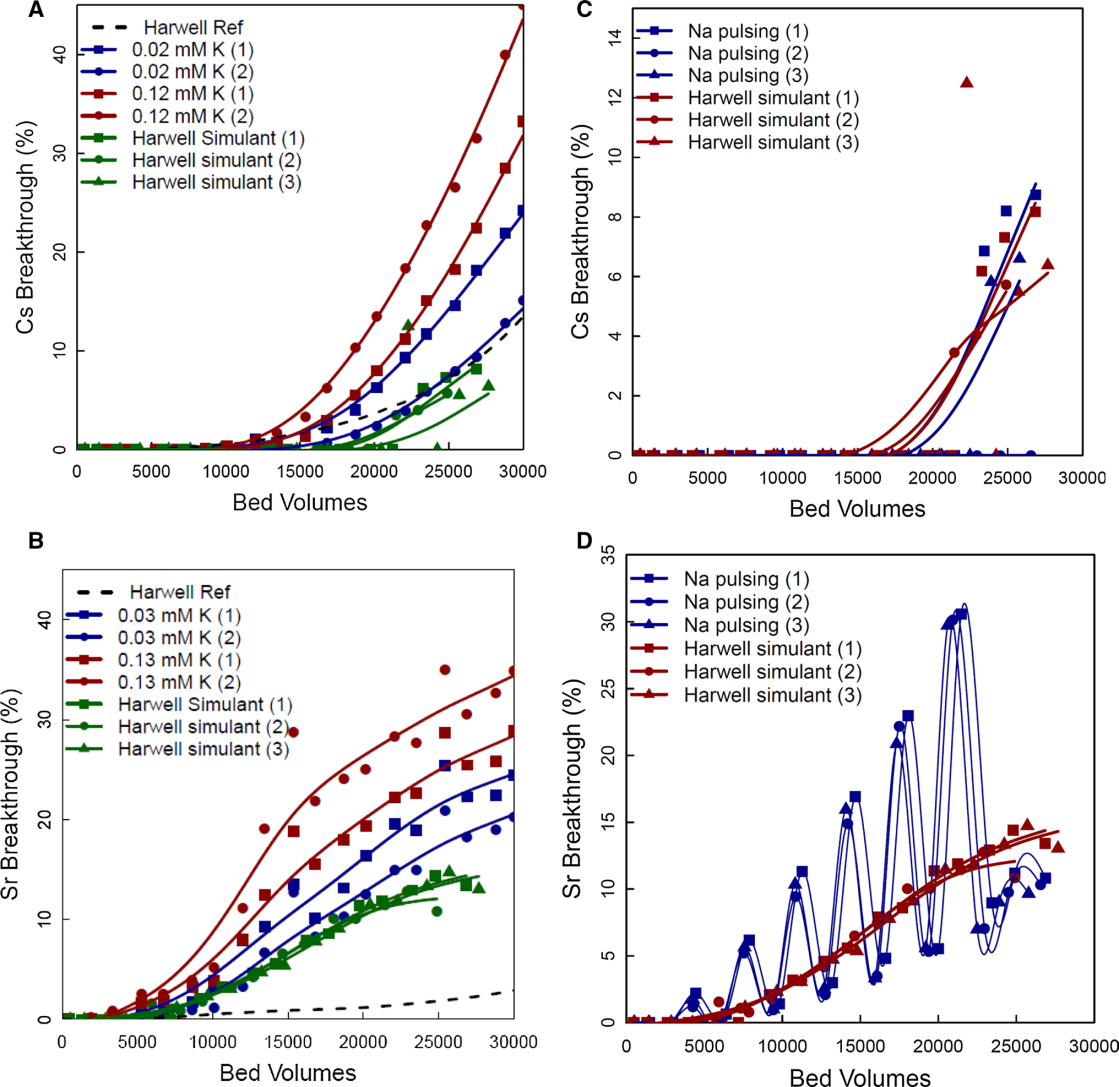
Effect of K^+^ on breakthrough of Cs^+^/Sr^2+^—left hand plots; Cs^+^/Sr^2+^ breakthrough in Na^+^ pulsing experiments—right hand plots

Breakthrough profiles generated with known concentrations of K^+^ are given for Cs^+^ and Sr^2+^ (Fig. 7a, b). Column experiments were also performed with a feed solution containing no added K^+^. For comparison, the Harwell Reference curves from Fig. 2a are also plotted in the figures.

Increasing the K^+^ concentration led to an expected reduction in capacity for Cs^+^ and Sr^2+^. As the K^+^ concentration was increased from 0.02 to 0.12 mM (1–5 ppm), the breakthrough profile was shifted further to the left. The number of BV required for a breakthrough of 5% illustrated the effect of K^+^ on the behaviour. For Cs^+^, 5% breakthrough occurred at: 23; 21 and 17.5 kBV, for 0, 0.02 and 0.12 mM K^+^, respectively. In the case of Sr^2+^, 5% breakthrough occurred at: 13, 11.5 and 9.5 kBV, respectively. Figure 7a, b shows the results of replicate measurements for both K^+^ concentrations (duplicates) and for the unaltered Harwell simulant (triplicates). For all systems, there was some variation from one individual column experiment to the next, but it is still possible to discern the effect of the K^+^ on breakthrough. There were significant differences between the Harwell simulant breakthrough curves recorded in the second phase experiment (green lines, Fig. 7a, b) and the original Harwell Reference Curves, particularly for Sr^2+^. Given the variation in performance across the Mud Hills deposit and even within the smaller mined area (Fig. 6), this was to be expected. Clearly, in these systems, it is important to compare results for the same sample of zeolite.

#### Effect of Na pulsing

With the commencement of legacy waste retrieval operations and decommissioning activities at Sellafield, changes in the composition of the feeds that will be treated by SIXEP are expected. It has been suggested that significant improvements in controlling the levels of competing ions entering SIXEP could be achieved by increasing the pH of the feed liquor, since that would result in the removal of soluble Mg, and potentially Sr, from the solution, as insoluble hydroxides. This would be expected to have a positive impact on performance. However, it would also result in increased Na concentrations in the SIXEP feed solutions.

Column trials were conducted to investigate the effect of pulsing the Na concentration, from a 100 ppm base-level (Harwell Reference simulant) to a 250 ppm (4.3–10.8 mM) ‘worse-case’ scenario, based on the maximum expected pH (12). The simulant with high Na concentration was based on the standard Harwell simulant, with an additional dose of Na ions, which was added as Na bicarbonate, since any additional NaOH added to the solutions would be converted to NaHCO_3_ in the carbonation tower, prior to the clinoptilolite columns. The high Na simulant was injected for approximately 3 days before returning to the original Harwell simulant for the remainder of each week. This regime was selected because of the ease of running the experimental trials and does not replicate a specific process at Sellafield. No Na pulsing was attempted during the first week of the trial to allow the system to stabilise and pulsing was terminated 2 weeks before the end of the experiment to assess how the system would respond to a return to the standard Harwell simulant.

The ^137^Cs breakthrough profiles for the Na pulsed columns are shown in Fig. 7c. Breakthrough profiles of the columns run with the standard Harwell simulant have also been included for comparison. There was good agreement between the triplicate experiments. The shape of the breakthrough profile was similar for each trial, although the onset of breakthrough varies slightly. There was little difference between the Cs^+^ breakthrough profiles observed for the Na pulsed columns and the Harwell simulant only columns. No significant breakthrough of ^137^Cs was observed in any of the trials until approximately 20 kBV of solution had been processed. The affinity of clinoptilolite is much greater for Cs than for Na, although it was expected that the increase in the Na ions might result in a greater competing common ion effect for Cs exchange. This was not observed in these trials. The Sr^2+^ breakthrough profiles for the Na pulsed columns are shown in Fig. 7d.

Increased discharges of Sr^2+^ from the column were observed during periods when the high Na simulant was being fed through the columns. The effect was reproducible, as the three replicate columns exhibited similar discharge profiles (Fig. 7d). The effect appears to be relatively short-lived, as the Sr^2+^ breakthrough in the pulsed columns returned to a level slightly below that observed for the Harwell simulant (low [Na^+^]) by the next sampling point after the simulant feed had been switched back to the low Na (Harwell) feed. This shows that the Na exchange reaction kinetics are relatively fast, and is consistent with the observations of Faghihian et al. [29]. However, total Sr^2+^ output from the Na^+^ pulsed columns was greater than for the non-pulsed columns by approximately 40% (based on the area under the curve).

The apparent improved performance, compared to that of the lower Na columns, when the flow of the high Na simulant was stopped could be attributed to a pre-conditioning effect on the clinoptilolite columns. The Na ions will effectively replace the other ions in the clinoptilolite. This increases the number of Na sites present on the clinoptilolite (i.e. an increased effective CEC), which will increase the likelihood of Sr^2+^ uptake once the Na concentration is reduced, and hence improve the removal of Sr. Beyond the fact that clinoptilolite shows a low selectivity for Na^+^ [4, 5, 32], it has been suggested that exchange for Na is quicker [27], and so in a system with a short residence time like this, a Na exchanged material should be more effective.

As the experiment progressed, the oscillations became more pronounced, as the total Sr^2+^ loading of the column increased, and so more Sr^2+^ was displaced when the Na concentration increased.

In the experiments reported here, the columns were pristine, i.e., “as mined” before a high Na feed was introduced. Given that the SIXEP plant operates two columns in series, it is expected that there will always be some activity associated with the lead bed. Therefore, some of the ions being removed by a high Na feed would be radio-isotopes. The extent of this effect would probably depend upon the loading state of the clinoptilolite prior to the increase in Na concentration.

It is important to contrast the response of the clinoptilolite sample used in the later pulsed Na experiments (Fig. 7c, d) with that of the original, Harwell sample (Fig. 4c, d). The uptake of Sr^2+^ by the clinoptilolite used in the second phase experiments (Fig. 7d) was more sensitive to competing Na ions than that observed during the first phase (Fig. 4d). As discussed above, the original sample performance represents the optimum for Sr^2+^, and for this sample, Na^+^ was not able to compete. The Sr^2+^ affinity of the sample used later is lower, and so Na^+^ can compete. Although divalent competitors are expected to be the strongest competitors based on structural arguments [17, 18], Um and Papelis [17] have suggested that direct competition with Na^+^ for exchange sites may be possible. The performance for Cs^+^ shows the opposite effect, with the original sample (Fig. 4c) more sensitive than the second phase sample (Fig. 7c).

## Modelling

There are a limited number of models of radionuclide interactions with clinoptilolite described in the literature. Valcke et al. [12, 13] developed a three-site model for predicting the binding of trace Sr^2+^ and Cs^+^ by clinoptilolite at equilibrium that could explain competition by Ca^2+^, Mg^2+^ and K^+^. Pabalan and Bertetti [33] successfully used a model, which was based on a Margules solid solution approach for the zeolite components and Pitzer equations for the activities of the ions in solution, to describe the equilibrium uptake of Sr^2+^.

Nikashina et al. [10] developed a model of Sr^2+^ interaction with clinoptilolite, which assumed that there were two distinct regions where radionuclide exchange could take place, the mesopores and the micropores. Initial transfer of metal ions to the mesopores was described with a kinetic equation. This was followed by slower diffusion into the micropores. The model was able to describe the results of column experiments including those where the flow was interrupted. Recently, Yin et al. [34] have used a kinetic sorption model, rather than ion exchange, to simulate the removal of ^90^Sr from contaminated water by clinoptilolite. The authors found that the apparent sorption rate constant was a function of the pore water velocity, rate constant decreasing as flow rate increased.

A coupled chemical transport model was developed to describe the behaviour of Cs^+^ and Sr^2+^ in SIXEP. The model was calibrated using the data from the original Harwell experiments only. Given the short residence times in the SIXEP column compared to the time required for equilibrium, a thermodynamic approach, such as those of Valcke et al. [12, 13] and Pabalan and Bertetti [33], were not appropriate. Instead, a kinetic ion exchange approach was adopted. The model assumes that the ionic concentration at a given column depth is independent of radial position. The model assumes that the columns are ‘well designed’, i.e., edge effects are not significant. Species are assumed to exist in one of two phases. They are either in the solution (mobile) or on the clinoptilolite (static). Ion exchange reactions represent the exchange between the mobile and static phases. To calculate the behaviour of the ions, the model splits the column into several discrete sections or nodes. Similarly, the total simulation time (in this case, the column run time) is split into several discrete time steps. At each node, and for each time step, a calculation is made of the ion exchange chemistry and the associated changes in solution concentration are calculated due to fluid flow and dispersion.

The flow of liquid through the column is pressure-driven incompressible fluid flow and is assumed to be determined entirely by the feed volumetric flow rate. There is an excluded volume due to the particles of clinoptilolite. This results in a higher face velocity for the fluid, scaled by the ratio of the total volume to the excluded volume. In addition to the concentration change at a given depth down the column due to fluid flow, there is also an axial dispersion of the concentration due to diffusion. This is modelled using the second derivative version of Fick’s Law [35]. The resulting change in concentration with time, due to fluid flow down the column and diffusion/dispersion is given by (2),

2 $$\frac{{dC_{i} }}{{{\text{d}}t}} = V_{\text{liq}} \frac{{dC_{{i}} }}{{{\text{d}}z}} - D_{i} \frac{{d^{2} C_{{i}} }}{{{\text{d}}z^{2} }}$$

3 $$\frac{{dC_{{{\text{liq,}}i,z}} }}{{{\text{d}}t}} = V_{\text{liq}} \frac{{dC_{{{\text{liq}},i,z}} }}{{{\text{d}}z}} - D_{i} \frac{{d^{2} C_{{{\text{liq}},i,z}} }}{{{\text{d}}z^{2} }}$$

where *C*_liq*i,z*_ is the concentration of component *i* in the liquid phase at depth *z, D*_*i*_ is the dispersion coefficient of component *i*, and *V*_liq_ is the face velocity of the liquid and is given by:

4 $$V_{\text{liq}} = \left( {\frac{\text{Feed Volumetric Flowrate}}{{{\text{Column Area}} \times {\text{Ratio of Total to Excluded Volume}}}}} \right)$$

The model was implemented within the gPROMS process modelling code, which is computationally efficient and robust, particularly with respect to solving partial differential equations.

The basis for the simulation of the ion exchange equilibrium is the law of mass action [5] (Dyer 2007). In this case, there is a model requirement for the clinoptilolite to be charge balanced at all times. It is assumed that there is no delay due to mass transfer within the exchanger phase, i.e. the surface exchange reaction is the rate limiting step. The model reactions are like the first reaction step in the model of Nikashina et al. [10]. The rate constants describing the ion exchange reactions are specific to each cation pair, as they depend on the specific interaction of each ion with the clinoptilolite, which will be a function of cation size and charge.

As an example, the exchange reaction that takes place between Na and Cs is (5):

5 $${\text{Na}}_{\text{ex}}^{ + } + {\text{Cs}}_{\text{aq}}^{ + } \leftrightarrow {\text{Na}}_{\text{aq}}^{ + } + {\text{Cs}}_{\text{ex}}^{ + }$$

where the subscript ‘ex’ indicates an ion bound to the clinoptilolite, and ‘aq’ an ion free in solution. A rate equation is defined for this reaction (6):

6 $$\frac{{- {\text{ d[Na}}_{\text{ex}}^{ + } ]}}{{{\text{d}}t}} = k_{\text{NaCs}} [{\text{Na}}_{\text{ex}}^{ + } ][{\text{Cs}}_{\text{aq}}^{ + } ] - k_{\text{CsNa}} [{\text{Na}}_{\text{aq}}^{ + } ][{\text{Cs}}_{\text{ex}}^{ + } ]$$

where *t* is the time, square brackets represent concentrations, and *k*_NaCs_ and *k*_CsNa_ are the forward and backward rate constants, respectively. The same approach was used describe Sr exchange for Na (7):

7 $$2 {\text{Na}}_{\text{ex}}^{ + } + {\text{Sr}}_{\text{aq}}^{ 2+ } \leftrightarrow 2 {\text{Na}}_{\text{aq}}^{ + } + {\text{Sr}}_{\text{ex}}^{ 2+ }$$

and:

8 $$\frac{{- {\text{ d[Na}}_{\text{ex}}^{ + } ]}}{{{\text{d}}t}} = k_{\text{NaSr}} [{\text{Na}}_{\text{ex}}^{ + } ]^{2} [{\text{Sr}}_{\text{aq}}^{2 + } ] - k_{\text{CsNa}} [{\text{Na}}_{\text{aq}}^{ + } ]^{2} [{\text{Sr}}_{\text{ex}}^{2 + } ]$$

where *t* is the time, square brackets represent concentrations, and *k*_NaSr_ and *k*_SrNa_ are the forward and backward rate constants, respectively.Thus, to calculate the concentration of ions in the liquid phase with column depth, taking account of ion exchange, an additional interaction term is included in (2) which is the negative of the above rate equations, giving (9):

9 $$\frac{{{\text{d}}C_{{{\text{liq}},i,z}} }}{{{\text{d}}t}} = V_{\text{liq}} \frac{{{\text{d}}C_{{{\text{liq}},i,z}} }}{{{\text{d}}z}} - D_{i} \frac{{{\text{d}}^{2} C_{{{\text{liq}},i,z}} }}{{{\text{d}}z^{2} }} - \frac{{{\text{d}}C_{{{\text{ex}},i,z}} }}{{{\text{d}}z}}$$

where *C*_ex*,i,z*_ is the concentration of component *i* on the ion exchanger at depth *z*.

Analogous equations were defined for all monovalent and divalent exchange reactions.

Using such simple ion exchange processes could give a reasonable fit to the Cs^+^ behaviour, but that to the Sr^2+^ was unrealistic. Given that Na^+^ is the dominant ion exchanged with the clinoptilolite and is in excess in solution, the number of ion exchange equations, and thus rate constants, to be fitted was reduced by the assumption that all ion exchange reactions are mediated by Na^+^. Rate constants were fitted using the parameter estimation facility within the gPROMS process modelling code.

**Fig. 8:**
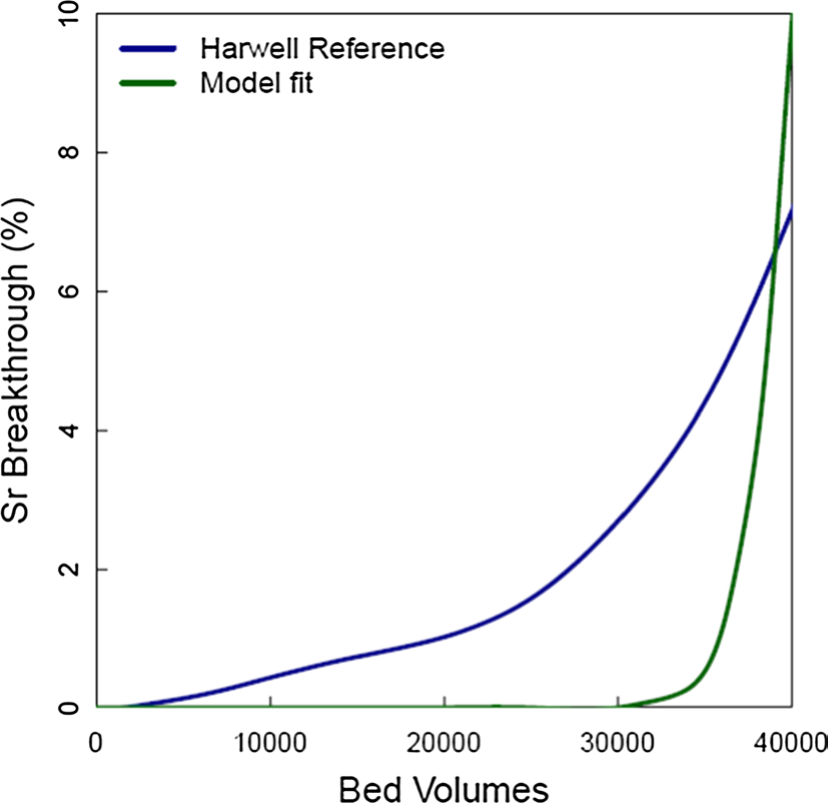
Original fit to Sr^2+^ data, excluding bicarbonate species

Figure 8 shows the fit obtained to the Sr^2+^ Harwell Reference data considering that Sr only interacts with the surface as Sr^2+^. It is clear from the figure that the shape of the breakthrough curve is completely different to that of the Harwell reference curve.

Ion selectivity in zeolites is governed by the anionic field (i.e. the concentration and spread of the aluminate units in the structure) and its pore dimensions. Clinoptilolite has a Si/Al ratio of 5 (a high silica concentration for this zeolite type), which gives it a low anionic field. This means that clinoptilolite is expected to be highly selective for cations with a low charge density (see above), and so the better performance of the clinoptilolite for Sr^2+^ over Cs^+^ shown in Fig. 2 is somewhat surprising.

**Fig. 9:**
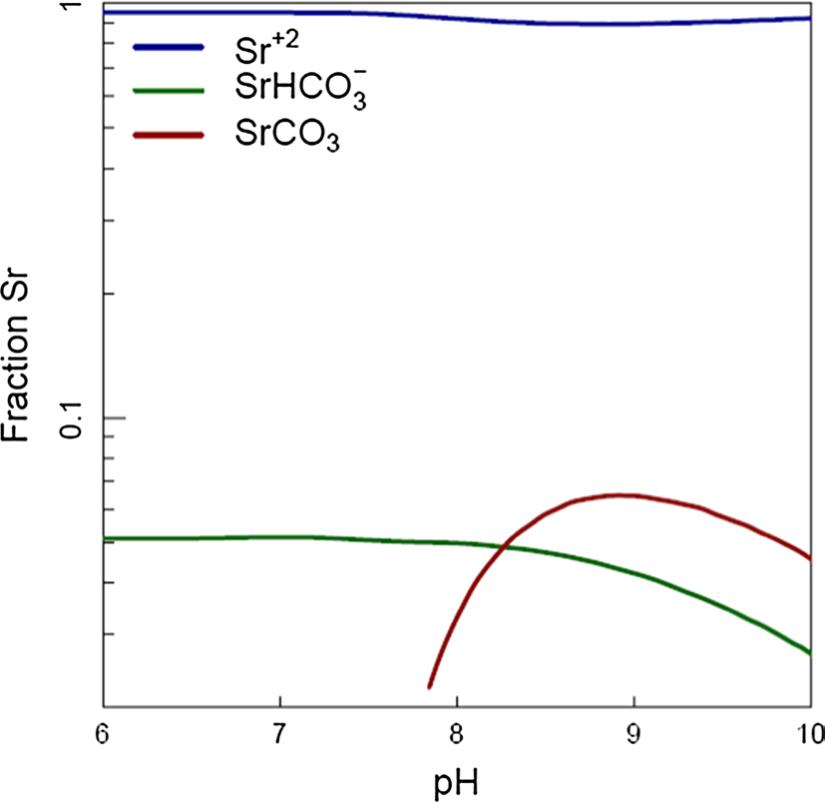
PHREEQC calculated speciation of Sr^2+^ in the clinoptilolite columns

The solution entering the SIXEP clinoptilolite columns (and the Harwell simulant) has a high background concentration of carbonate/bicarbonate, due to the neutralisation of the alkali in the solution in the carbonation tower. Bicarbonate will react with the divalent Group II cations in the following way (9):

10 $$X_{\text{aq}}^{2 + } + {\text{HCO}}_{{3({\text{aq}})}}^{ - } \leftrightarrow X{\text{HCO}}_{{3({\text{aq}})}}^{ + }$$

where *X* is a Group II cation. The PHREEQC equilibrium thermodynamic speciation modelling code [36] was used to calculate the speciation in the Harwell simulant (and SIXEP) column feeds. Figure 9 shows the predicted Sr^2+^ speciation following the carbonation tower in SIXEP and in the Harwell simulant solution. The SrHCO_3_^+^ species is present in quantities that are significant, although the free Sr^2+^ ion is still the dominant form in solution. The hydrolysis species, [Sr(OH)]^+^, only accounted for a very small fraction of total Sr^2+^ (< 0.1%).

The effective size and charge density of a [SrHCO_3_]^+^ cationic complex could be a better match for clinoptilolite, and this could be the reason for the improved Sr^2+^ performance of the material in the presence of CO_3_^2−^/HCO_3_^−^ [25, 26]. The rate of the ion exchange reaction for the complex may be favourable compared to that of the divalent species.

To test whether HCO_3_^−^ was influencing the interaction of Sr^2+^ with the clinoptilolite in SIXEP, batch experiments were performed to determine the distribution coefficients (K_d_) of Sr^2+^ for a K^+^ exchanged clinoptilolite in KNO_3_ solutions in the concentration range of 0.01–1 M and in the presence and absence of HCO_3_^−^ (1 mM). The results are shown in Table 2. An increase was observed in the presence of HCO_3_^−^.

**Table 2:** Distribution coefficients (*K*_*d*_) for Sr^2+^ with *K* exchanged clinoptilolite in KNO_3_ solutions, with and without bicarbonate (1 mM)

[KNO_3_] (M)	Sr^2+^ *K*_*d*_ (mL g^−1^) No bicarbonate	Sr^2+^ *K*_*d*_ (mL g^−1^) With bicarbonate (1 mM)
0.01	540	980
0.1	10	35
1	0.1	17

Therefore, the model was revised to allow exchange of HCO_3_^−^ complexes. It was found that the best fits to the Sr data were obtained using a combination of rate equations for exchange of Sr^2+^ and SrHCO_3_^+^, assuming a distribution of [Sr^2+^]/[SrHCO_3_^+^] ratio of 18.6:1, obtained using PHREEQC speciation calculations (Fig. 9).

Given the short residence time in the column, it seems likely that the majority of ion exchange happens at the surface of the clinoptilolite particles during column operation, and thus the diffusivity cannot be used to deduce the mechanism in this case. It is unclear whether a SrHCO_3_^+^ complex would exist within the clinoptilolite framework. The structural studies of O’Day [18] and Um and Papelis [17] were not performed in systems of high carbonate, and so the lack of bicarbonate incorporation there does not preclude that this takes place in SIXEP. Those studies do show that some coordinated water is retained when Sr^2+^ is incorporated, and so the ion does have vacant coordination sites. If SrHCO_3_^+^ is initially sorbed, once it enters the framework it may dissociate to give Sr^2+^ and HCO_3_^−^. As surface ion exchange dominates in the column, the same kinetics would be observed, whether or not the bicarbonate complex dissociates. However, if the complex does dissociate in the framework, then in the long-term, exchange deeper within the particles and the diffusion and the kinetics of Sr behaviour would be dominated by the properties of the divalent cation.

It should be noted that there are several mechanisms that could lead to the observed Sr^2+^ kinetics, for example, a mass transfer process that is not limited by film diffusion, such as surface sorption from a concentrated solution. The uptake of the bicarbonate complex can explain the column data and is consistent with other observations. However, the observed kinetics may be due to a combination of different processes that are currently unknown. Direct evidence for the bicarbonate mechanism is not available in the literature. Fondeur et al. [37] did find that increasing carbonate concentration increased the Cs^+^ K_d_ for the ion exchanger, IONSIV. However, the authors attributed that to a decrease in the activity of Na^+^ associated with carbonate addition, which reduced competition with Cs^+^ for exchange sites. Elizondo et al. [38] observed an increase in Sr^2+^ K_d_ for clinoptilolite from 1200 to 3700 mL/g as pH increased from 7 to 8. Their experiments do not appear to have used an inert atmosphere, and so dissolved carbonate concentration would be expected to increase with pH, although the increase could be associated with the change in pH. Smiciklas et al. [39] also observed that the uptake of Sr^2+^ and Cs^+^ by clinoptilolite increased with pH.

**Fig. 10:**
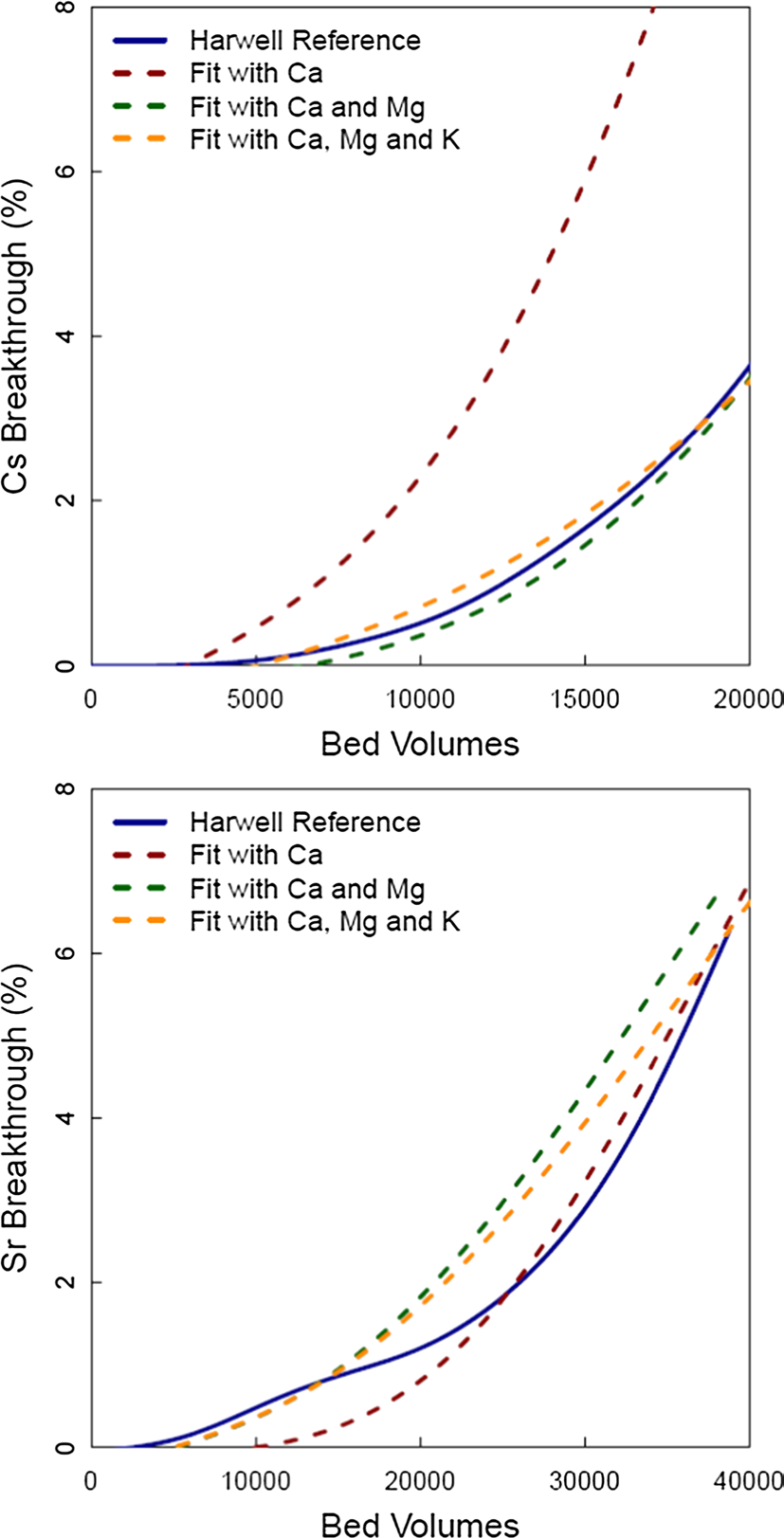
Model fit to Harwell reference Cs^+^/Sr^2+^ data

The model incorporating bicarbonate complexes was applied to the original Harwell column data. Figure 10 shows the model fits to the Harwell Reference curves, and Table 3 shows the best fit values of the rate constants. The level of uncertainty on the rate constants could not be calculated directly due to a lack of repeat experiments in the data source used. The value for dispersion was set to 900 dm^2^/day. The figure shows the results of calculations including competition from: Ca^2+^ only; Ca^2+^ and Mg^2+^; Ca^2+^, Mg^2+^ and K^+^. Considering the Cs^+^ data, it was clear that competition from both divalent cations was important, because significant improvements were achieved by successively including their effects. This was not surprising given the effect that these ions were found to have in the column tests (Figs. 3 and 4). The addition of K^+^ to the model made less of a difference. For Sr^2+^, the fit to the data was less good than for Cs^+^, but represented a clear improvement on the calculation excluding bicarbonate complexes (Fig. 8).

**Table 3:** Forward and backward rate constants (/s) for the ion exchange of Na in clinoptilolite with various ions in solution

	Backward	Forward
Cs^+^	4.38 × 10^−9^	1.15 × 10^−5^
Sr^2+^	9.17 × 10^−17^	1.86 × 10^−14^
SrHCO_3_^+^	1.46 × 10^−8^	9.17 × 10^−7^
Ca^2+^	5.87 × 10^−17^	1.50 × 10^−16^
Mg^2+^	1.43 × 10^−16^	6.27 × 10^−17^
MgHCO_3_^+^	2.29 × 10^−8^	5.42 × 10^−7^
K^+^	6.25 × 10^−10^	1.38 × 10^−6^

An estimate of parameter uncertainty has been calculated by re-running parameter estimation routines with different combinations of experiments and examining the variability of the predicted parameter values.

**Fig. 11:**
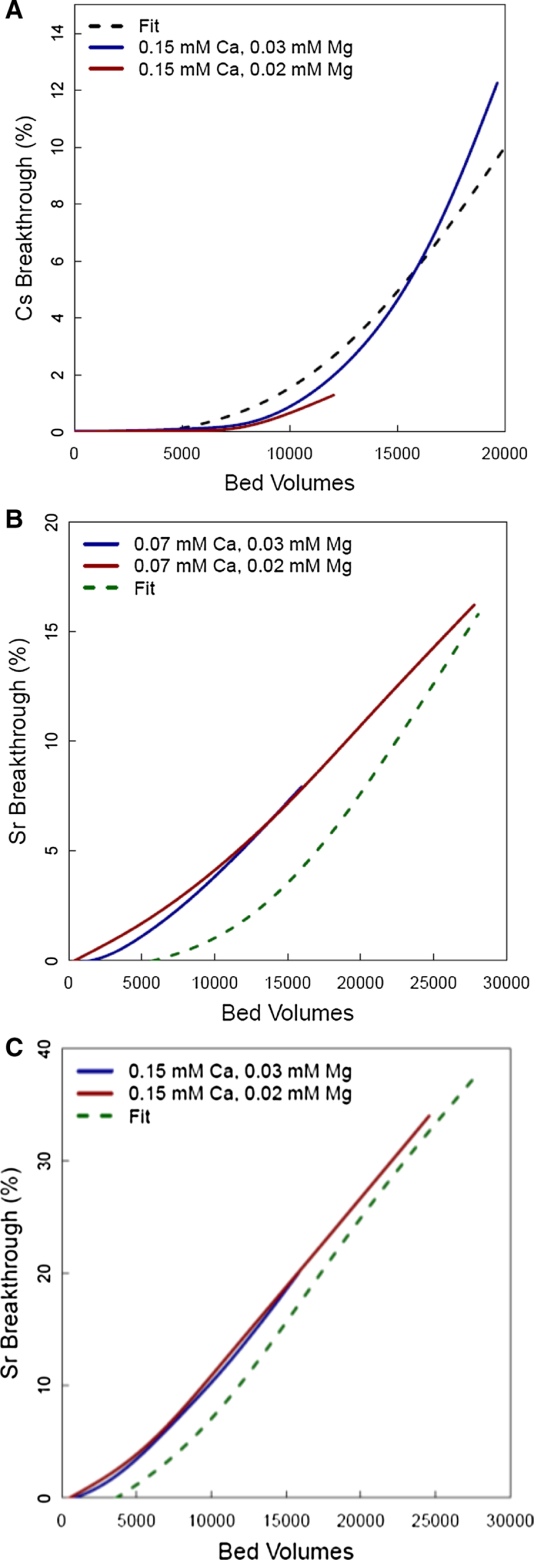
Ion exchange model fits ([Mg^2+^] = 0.02 mM (0.6 ppm)/0.03 mM (0.7 ppm)): **a** Cs^+^ data with [Ca^2+^] = 0.15 mM (6 ppm); **b** Sr^2+^ data with [Ca^2+^] = 0.07 mM (3 ppm); **c** Sr^2+^ data with 0.15 mM (6 ppm)

**Fig. 12:**
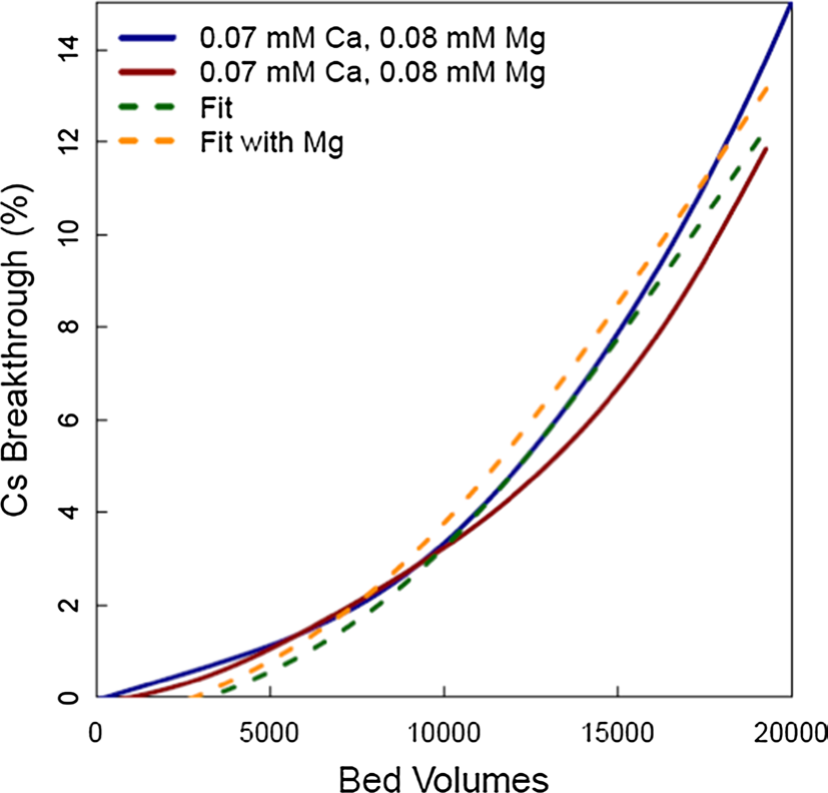
Cs^+^ ion exchange model fit with [Mg^2+^] = 0.08 mM (2 ppm)

Figure 11a shows the fit of the model to some of the other Cs^+^ Harwell data ([Ca] = 6 ppm = 0.15 mM). The fit was reasonable, given the inherent variability in the column data. Figure 11b, c shows the fit to the Sr^2+^ Harwell data ([Ca] = 3 and 6 ppm = 0.08–0.15 mM). Although the height of the breakthrough profiles could be reproduced with the modified rate constants, the shape of the experimental curves (straight line vs. curved profile in the modelled results) could not. At this stage, it cannot be attributed to any specific factors. The difference in shape between experiment and model prediction could be due to several possible mechanisms: (1) there are additional chemical reactions other than ion exchange at play (e.g. (co-)precipitation, surface sorption or ion exchange chromatography of colloidal material); (2) a difference in flow patterns between the different experiments (dispersion, flow rate, channelling or other edge effects); (3) variability in the clinoptilolite material. Figure 12 shows the fit to the Cs^+^ Harwell data for a Mg^2+^ concentration of 2 ppm (0.08 mM). As for the effect of enhanced Ca^2+^, the fit was reasonably good.

All transport models are to some extent semi-empirical, and the model described here is a simplified description of a complex system. However, it has been used successfully for over a decade to predict the performance of the ion exchange columns in SIXEP, and so it is effective as a model for plant support purposes. The sensitivity of the model to feed flow rates and ion concentrations has also recently been evaluated using Artificial Neural Networks [U. Oparaji et al./Neural Networks 96 (2017) 80–90].

## Conclusions

The column experiments have shown that clinoptilolite can be effective in removing Cs^+^ and Sr^2+^ ions from aqueous solutions that are simulants of the feed to the SIXEP plant. Although Ca^2+^, Mg^2+^, K^+^ and Na^+^ have all been shown to compete with Cs^+^ and Sr^2+^, at the concentrations in the SIXEP feed solutions, the performance was sufficient to control Cs^+^ and Sr^2+^ concentrations in the effluents. Higher concentrations of these competing ions could result in reduced removal of Cs^+^ and Sr^2+^, and consequently more frequent changing of the ion exchange beds. For Ca^2+^, Mg^2+^ and K^+^, concentrations much less than 1 mM were sufficient to reduce performance significantly, and so it is important to control the concentrations of these species in the SIXEP feed solutions carefully. The clinoptilolite has a low selectivity for Na ions, and concentrations of several millimolar are required to influence performance, although there is a high background concentration of Na^+^ in the feed solutions. One suggested method for the control of Ca^2+^ and Mg^2+^ is an increase in solution pH, via the addition of Na hydroxide. Although the associated reduction in the solubility of the Group II ions would benefit the performance, the increase in Na^+^ concentration would have the opposite effect, and so there will be an optimum pH for performance, which is likely to be different for Cs^+^ and Sr^2+^.

The experiments have shown that there is very significant variation in clinoptilolite performance from sample to sample, even from the same formation. The SIXEP plant has been operating for 3 decades, and has succeeded in reducing the discharges of ^134/137^Cs and ^89/90^Sr to the Irish Sea during that time. Although the performance of the clinoptilolite is variable, even within a small subsection of a single mine, by monitoring of the effluent from the plant, it is possible to control the discharges.

The model described in this paper can simulate the behaviour of the clinoptilolite in laboratory column experiments. It has also been applied to the performance of the plant itself, and it can also successfully simulate its performance. The good performance of the clinoptilolite in removing Sr^2+^ from solution in the laboratory and on plant was somewhat surprising, given the zeolite Si/Al ratio. It seems that the formation of metal bicarbonate ions of the divalent Group II ions may play an important role in the success of the ion exchange process, although more work would be required to demonstrate that conclusively.

This work shows that a combination of laboratory scale experiments and coupled chemical transport modelling can be used to design and optimise the performance of a successful nuclear effluent treatment plant. As the feed solutions to the plant change during the decommissioning and remediation of the Sellafield site, the understanding of the mechanisms and processes controlling the behaviour of radioisotopes reported here will play an important role in maintaining low discharge rates in the future.

## Electronic supplementary material

Below is the link to the electronic supplementary material.

Supplementary material 1 (DOCX 1127 kb)
