# Supplementary material for: The use of columns of the zeolite clinoptilolite in the remediation of aqueous nuclear waste streams
Source: J Radioanal Nucl Chem. 2018 Nov 22;318(3):2473–91. doi: 10.1007/s10967-018-6329-8 (PMC6267155; doi:10.1007/s10967-018-6329-8)
Supplement: Supplementary file 1 — Supplementary material 1 (DOCX 1127 kb) [file 10967_2018_6329_MOESM1_ESM.docx]

**Supporting Information (1)**

**Fig. A1:** Schematic diagram of column experimental rig: 1. Economy drive pump for transferring liquor from non-active simulant tank (2) located outside the fumehood to the radioactive dosing tank located inside the fumehood (4); 2. Non-active simulant tank; 3. 2 way valve; 4. Radioactive dosing tank – where the dosing of the non-active simulant with radiotracer occurs; 5. Economy drive pump for transferring dosed liquor from dosing tank (4) to continuous feeding tank for the columns (8). 6. 3-Way Valve – allows a sample of the feed liquor to be taken from each batch; 7. Level Sensor; 8. Continuous column feed tank; 9. Column feed precision drive pump; 10. Leak detector pad; 11. Drip tray; 12. Stainless steel pump heads for LS14 tubing; 13. 3 way valves; 14. Column assembly; 15. Drain; 16. Liqui Sense detector – for detection of leaks and monitoring level of liquor within the continuous feed tank.

**Fig. A2:** Photograph of column study rig.

**Fig. A3:** Schematic diagram of columns

**Fig. A4:** Photograph of packed column.
